# Neutrophil and mononuclear leukocyte pathways and upstream regulators revealed by serum proteomics of adult and juvenile dermatomyositis

**DOI:** 10.1186/s13075-024-03421-7

**Published:** 2024-11-11

**Authors:** A. Clare Sparling, James M. Ward, Kakali Sarkar, Adam Schiffenbauer, Payam Noroozi Farhadi, Michael A. Smith, Saifur Rahman, Kamelia Zerrouki, Frederick W. Miller, Jian-Liang Li, Kerry A. Casey, Lisa G. Rider

**Affiliations:** 1 Environmental Autoimmunity Group, Clinical Research Branch, National Institute of Environmental Health Sciences, National Institutes of Health, Building 10, CRC Rm 6-5700, MSC 1301 10 Center Drive, Bethesda, MD 20892-1301 USA; 2 Integrative Bioinformatics Support Group, National Institute of Environmental Health Sciences, National Institutes of Health, Research Triangle Park, Durham, NC USA; 3 BioPharmaceuticals R&D, AstraZeneca, Gaithersburg, MD USA

**Keywords:** Dermatomyositis, Juvenile dermatomyositis, Biomarkers, Disease activity, Neutrophils, Monocytes, Multiplex proteomics

## Abstract

**Objectives:**

Serum protein abundance was assessed in adult and juvenile dermatomyositis (DM and JDM) patients to determine differentially regulated proteins, altered pathways, and candidate disease activity biomarkers.

**Methods:**

Serum protein expression from 17 active adult DM and JDM patients each was compared to matched, healthy control subjects by a multiplex immunoassay. Pathway analysis and protein clustering of the differentially regulated proteins were examined to assess underlying mechanisms. Candidate disease activity biomarkers were identified by correlating protein expression with disease activity measures.

**Results:**

Seventy-eight of 172 proteins were differentially expressed in the sera of DM and JDM patients compared to healthy controls. Forty-eight proteins were differentially expressed in DM, 32 proteins in JDM, and 14 proteins in both DM and JDM. Twelve additional differentially expressed proteins were identified after combining the DM and JDM cohorts. C-X-C motif chemokine ligand 10 (CXCL10) was the most strongly upregulated protein in both DM and JDM sera. Other highly upregulated proteins in DM included S100 calcium binding protein A12 (S100A12), CXCL9, and nicotinamide phosphoribosyltransferase (NAMPT), while highly upregulated proteins in JDM included matrix metallopeptidase 3 (MMP3), growth differentiation factor 15 (GDF15), and von Willebrand factor (vWF). Pathway analysis indicated that phosphoinositide 3-kinase (PI3K), p38 mitogen-activated protein kinase (MAPK), and toll-like receptor 7 (TLR7) signaling were activated in DM and JDM. Additional pathways specific to DM or JDM were identified. A protein cluster associated with neutrophils and mononuclear leukocytes and a cluster of interferon-associated proteins were observed in both DM and JDM. Twenty-two proteins in DM and 24 proteins in JDM sera correlated with global, muscle, and/or skin disease activity. Seven proteins correlated with disease activity measures in both DM and JDM sera. IL-1 receptor like 1 (IL1RL1) emerged as a candidate global disease activity biomarker in DM and JDM.

**Conclusion:**

Coordinate analysis of protein expression in DM and JDM patient sera by a multiplex immunoassay validated previous gene expression studies and identified novel dysregulated proteins, altered signaling pathways, and candidate disease activity biomarkers. These findings may further inform the assessment of DM and JDM patients and aid in the identification of potential therapeutic targets.

**Supplementary Information:**

The online version contains supplementary material available at 10.1186/s13075-024-03421-7.

## Introduction

Myositis is a heterogeneous group of rare systemic autoimmune diseases characterized by chronic inflammation in skeletal muscle [1]. It is thought to be caused by a combination of genetic and environmental factors. Dermatomyositis in adults and children (DM, JDM) has characteristic cutaneous features and can be subclassified by myositis-specific autoantibodies (MSAs) [1]. While both DM and JDM patients exhibit photosensitive rashes, proximal weakness, and other similar illness features, they differ in certain clinical features. For example, DM is associated with an increased risk of interstitial lung disease and cancer, while calcinosis is more common in JDM patients [1, 2].

Gene and protein expression, histopathology, and other studies of affected tissues have provided insight into the molecular mechanisms underlying DM and JDM. Adaptive immune cells, such as B cells, CD4 + and CD8 + T cells, and innate immune cells, such as dendritic cells (DCs), macrophages, and neutrophils, have been shown to infiltrate DM and JDM muscle and skin tissues [3–5]. A type I and II interferon (IFN) gene signature has been widely reported in the peripheral blood and affected skin and muscle of DM and JDM patients [4, 6–8]. Recent studies have demonstrated upregulated neutrophil degranulation pathways in DM and JDM, and neutrophil extracellular traps (NETs) are thought to directly contribute to tissue damage [9–11].

Due to the heterogeneity and complexity of DM and JDM, reliable biomarkers are needed to inform diagnosis, prognosis, and response to treatment [12]. Serum muscle enzymes, including creatine kinase (CK), aldolase, and lactate dehydrogenase (LDH), are commonly used as disease activity biomarkers in DM and JDM. However, these enzymes often fail to correlate with muscle weakness and rashes present during chronically active disease [13]. Thus, additional measures are needed to monitor disease activity. Further, few studies have directly compared DM and JDM disease activity biomarkers or used a systematic protein platform that examines multiple proteins simultaneously [12, 14].

In this study, a broad protein immunoassay examined patient sera from active DM and JDM patients undergoing treatment to identify dysregulated proteins, altered pathways, and candidate disease activity biomarkers. Similarities and differences in these proteins, pathways, and biomarkers between DM and JDM patients were also investigated.

## Materials/methods

### Patients and sample collection

Patients (17 DM, 17 JDM) that met definite European Alliance of Associations for Rheumatology – American College of Rheumatology (EULAR-ACR) criteria were enrolled in an institutional review board approved myositis natural history study (NCT00059748) at the National Institutes of Health (NIH) Clinical Center [15]. Seventeen adult healthy controls were selected from the internal donor program approved by the MedImmune institutional review board and matched to DM patients by sex and age within a decade; race/ethnicity was unavailable. Fifteen pediatric healthy controls were enrolled at the NIH Clinical Center and matched to JDM patients by sex, age within six years, and race/ethnicity. Two MedImmune healthy control subjects were also matched to JDM patients by sex and age within a decade. International Myositis Assessment and Clinical Studies (IMACS) Group and Paediatric Rheumatology INternational Trials Organisation (PRINTO) core set measures were used to assess disease activity in DM and JDM patients [16, 17]. Autoantibodies were tested via immunoprecipitation (IP), immunoblotting (IB), IP-IB, and enzyme-linked immunosorbent assay methods [18–21]. Sera were collected and stored at -80 °C. The majority of these patients were included in a prior study on transcriptomics by microarray and proteomics by SomaLogic [11].

### Protein measurement and expression analysis

Serum protein measurements of 282 proteins were determined by a quantitative multiplex immunoassay using the Human DiscoveryMAP v. 3.3 Multi-Analyte Panel (Myriad Rules-Based Medicine [RBM], Austin, TX). Protein concentrations were log2-transformed. One-hundred ten proteins had undetectable serum concentrations in some patients and were excluded from the study. Differentially expressed proteins were validated with SOMAscan Assay v3.2 (SomaLogic) aptamer-based DNA probes, as previously described [11].

DM and JDM patients and matched controls were grouped into adult, juvenile, or combined (adult and juvenile) categories for analysis. Normally distributed proteins were defined through a Shapiro-Wilk test on the residuals of the log-transformed data, with *p* < 0.05. For normally distributed proteins, a two-sided, unpaired moderated t-test was performed using limma (version 3.40.6) [16, 22]. A blocking factor for patient cohort was included in the combined analysis of DM and JDM patients. A two-sided Mann-Whitney test was used for non-normally distributed proteins. Significantly altered protein concentrations were defined as Benjamini-Hochberg (BH) adjusted *p* < 0.1 and |fold change| > 1.1 compared to control subjects [23]. P-values were adjusted independently for the limma and Mann-Whitney analyses. Entrez gene symbols were substituted for RBM protein identifiers to allow comparison across platforms using UniProt’s ID mapping tool. Heatmaps were created using ComplexHeatmap in R [24]. The superset of 78 dysregulated proteins across these comparisons was used for subsequent analyses.

### Protein clusters

Spearman correlations were calculated among the 78 dysregulated proteins, separately in DM and JDM patients. Correlations among proteins with *p* < 0.05 and |Spearman’s rank correlation coefficient| > 0.4 were plotted on a heatmap. Correlations that did not meet these thresholds were assigned a value of “NA,” so that such proteins did not influence the protein clustering. The two largest protein clusters in each heatmap were further examined in additional heatmaps. Cell type-specific protein expression profiles obtained from ProteinAtlas.org were analyzed for the largest protein cluster [11]. Potential associations between proteins in the second largest protein cluster and IFNs were determined using the Interferome software (version 2.0) [25].

### Pathway enrichment

Differentially regulated proteins, 48 in DM and 32 in JDM, were independently tested for enrichment of canonical pathways and biological functions using Ingenuity Pathway Analysis (IPA) (version 23.0). The whole genome was used as background, only human data was included, and the threshold for reporting significance was an adjusted *p* < 0.05 [26]. Predicted upstream regulators of the differentially regulated proteins were determined, and those with *p* < 0.05 and |activation z-score| > 1.5 were reported. A regulatory effects diagram was created for DM using the IPA software to examine upstream regulators with strong interconnectedness between functions and molecules [26].

### Disease activity correlations

Spearman rank correlations were calculated between the 78 dysregulated proteins and disease activity measures for DM patients, and separately for JDM patients. Measures of global activity included total scores for the Disease Activity Score (DAS) and the Myositis Disease Activity Assessment Tool (MDAAT), as well as Physician (MD) Global Disease Activity [27]. Manual Muscle Testing of 26 proximal, distal, and axial muscles (MMT26), Childhood Myositis Assessment Scale (CMAS), and DAS Muscle were used as measures of muscle disease activity [27]. DAS Skin and MDAAT Cutaneous visual analog scale (VAS) were used as measures of skin disease activity [27]. Correlation coefficients for MMT26 and CMAS were multiplied by negative one for consistent directionality. Correlations of proteins measured by the RBM platform with serum levels of muscle enzymes were also examined, including LDH, aldolase, and CK. Correlations with *p* < 0.05 and |Spearman’s rank correlation coefficient| > 0.4 were clustered in a heatmap, with correlations below these thresholds assigned a value of “NA”.

## Results

Patient characteristics between the DM and JDM patients were similar in terms of race and ethnicity, MSAs, disease duration, disease activity, and daily glucocorticoid dose (Table 1). All patients, except for one with DM, were receiving treatment during the study. JDM patients were receiving a greater number of immunosuppressive or immunomodulatory medications compared to DM patients. Medications included immunosuppressive drugs, such as prednisone and methotrexate, and biologics such as rituximab and intravenous immunoglobulin (IVIg) (Table 1).

**Table 1:** Clinical and demographic characteristics of adult and juvenile dermatomyositis patients

Feature	DM (*n* = 17)	JDM (*n* = 17)
	Median [Q1, Q3] or *n* (%)	Median [Q1, Q3] or *n* (%)
Age (years)	52.6 [49.9, 61.7]*	13.3 [9.4, 16.6]*
Female subjects	15 (88.2%)	9 (52.9%)
Race and ethnicity		
African American	4 (23.5%)	2 (11.8%)
Asian	0 (0.0%)	2 (11.8%)
Hispanic	0 (0.0%)	2 (11.8%)
White	13 (76.5%)	11 (64.7%)
Myositis-specific autoantibodies		
NXP2	5 (29.4%)	6 (35.3%)
TIF-1	5 (29.4%)	3 (17.6%)
MDA5	1 (5.9%)	5 (29.4%)
Mi2	1 (5.9%)	0 (0.0%)
Jo-1	3 (17.6%)	0 (0.0%)
None	3 (17.6%)	3 (17.6%)
Disease duration (years)	1.5 [1.0, 10.0]	3.5 [0.5, 5.8]
Daily glucocorticoid dose (mg/kg/day)	0.06 [0.04, 0.26]	0.23 [0.0, 0.51]
Additional immune therapies†	1.0 [1.0, 2.0]*	3.0 [2.0, 4.0]*
Disease Activity Measures (potential range)		
Global Activity		
MD Global (0–10 cm VAS)	1.9 [0.4, 6.3]	1.9 [1.6, 4.2]
MDAAT Total (0–52)	18.0 [11.5, 27.0]	10.0 [8.0, 17.5]
DAS Total (0–20)	11.0 [9.0, 13.5]	10.5 [8.0, 13.5]
Muscle Activity		
Total MMT (0-260)	236.0 [201.0, 245.5] ‡	246.0 [234.5, 260.0] ‡
CMAS (0–52)	27.0 [0.0, 48.5]	46.0 [37.5, 48.0]
DAS Muscle (0–11)	5.0 [3.8, 8.0]	4.5 [2.8, 8.0]
Skin Activity		
DAS Skin (0–9)	6.0 [4.5, 7.0]	5.0 [5.0, 6.0]
MDAAT Cutaneous (0–10 cm VAS)	2.1 [0.3, 5.0]	2.4 [1.3, 4.2]
Enzymes		
Aldolase (1–7 U/L)	4.5 [5.0, 8.7]	6.3 [6.0, 8.8]
CK (30–252 U/L)	113 [70, 163]	87 [65, 114]
LDH (125–226 U/L)	225 [186, 296]	184 [151, 210]

* DM v. JDM *p* < 0.0001

† Additional immunotherapies received at study enrollment (#DM, #JDM): methylprednisolone (0, 9), methotrexate [9, 10], hydroxychloroquine [4, 9], azathioprine (1, 0), cyclosporin (1, 0), mycophenolate [3, 6], tacrolimus (0, 1), cyclophosphamide (0, 1), IV gammaglobulin (IVIg) [4, 11], rituximab [1], and tofacitinib (0, 1)

‡ DM v. JDM *p* < 0.05

Abbreviations: DM, dermatomyositis; JDM, juvenile dermatomyositis; Q1, first quartile; Q3, third quartile; MD, Physician; VAS, visual analog scale; MDAAT, Myositis Disease Activity Assessment Tool; DAS, Disease Activity Score; MMT, Manual Muscle Testing; CMAS, Childhood Myositis Assessment Scale; CK, creatine kinase; LDH, lactate dehydrogenase

### Differentially expressed proteins in DM and JDM

**Fig. 1:**
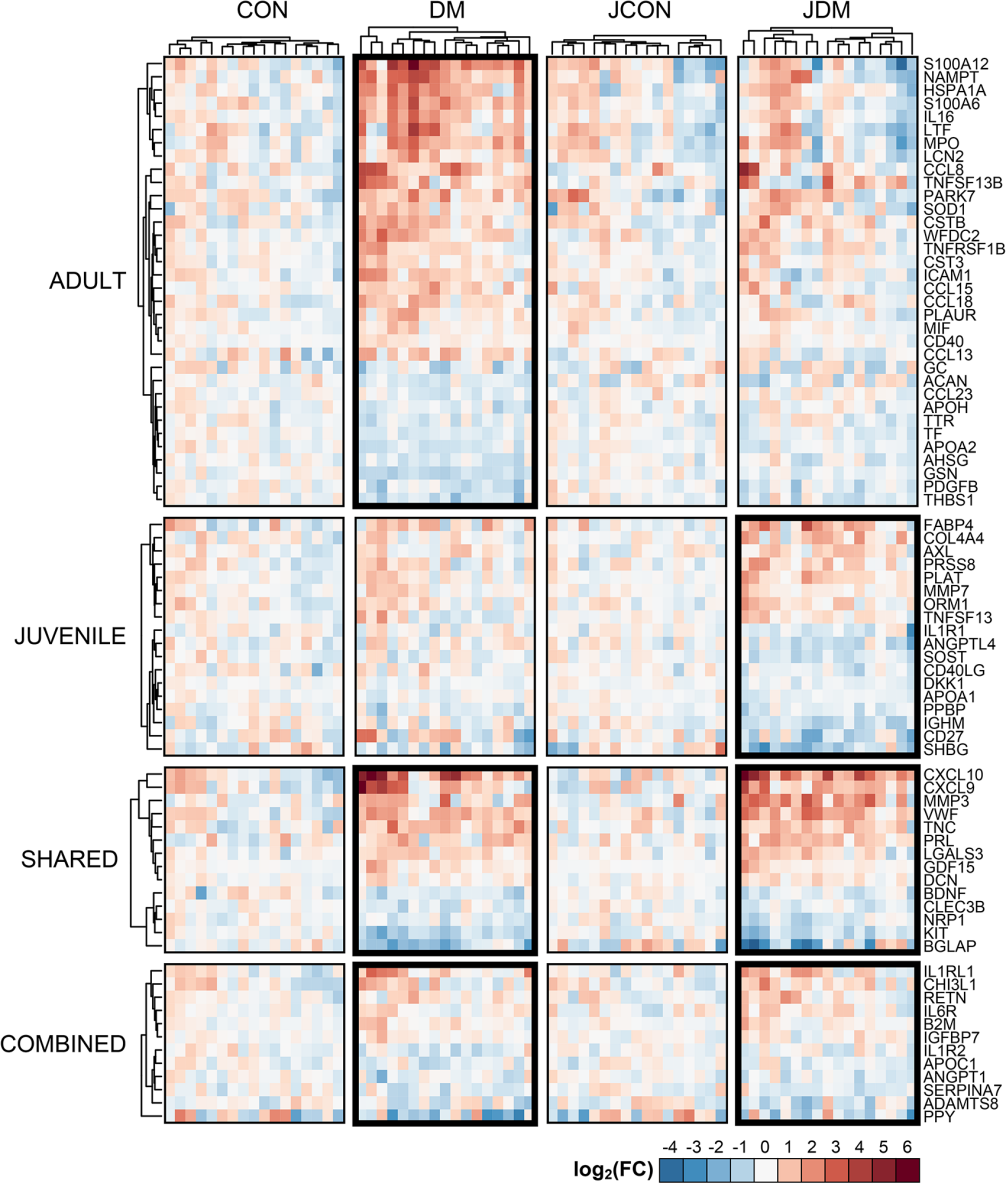
Differentially expressed proteins in adult dermatomyositis (DM) and juvenile dermatomyositis (JDM) patients compared to healthy controls. Red indicates upregulation, blue indicates down regulation, and white indicates no change. The row groupings indicate the proteins that had significantly different expression between DM or JDM patients and healthy controls in the adult analysis (adult), the juvenile analysis (juvenile), both the adult and juvenile analyses (shared), as well as the new proteins that arose when the adult and juvenile datasets were combined (combined). Abbreviations: CON, adult control; DM, adult dermatomyositis; JCON, juvenile control; JDM, juvenile dermatomyositis; FC, fold change

Forty-eight and 32 serum proteins were differentially expressed in DM and in JDM patients, respectively. Of these proteins, 14 were differentially expressed in both DM and JDM patients (Supplementary Fig. 1). The combined cohort with both the adult and juvenile datasets identified 12 additional proteins that were differentially expressed between DM/JDM patients and healthy controls. In total, 78 serum proteins were differentially expressed in DM and JDM patients (Fig. 1). SomaLogic was used to validate the differentially expressed proteins. Of the 48 and 32 differentially expressed proteins in DM and JDM, respectively, 39 and 22 were present in the SomaLogic assay, and 17 and 9 were identified as differentially expressed in the SomaLogic assay in DM and JDM (43.6% and 40.9% confirmation, respectively). For DM, 31 of the 39 shared proteins had a concordant fold-change direction between the SomaLogic and RBM platforms (81.6% confirmation). For JDM, 20 of the 22 shared proteins had a concordant fold-change direction (90.9% confirmation).

The C-X-C motif chemokine ligand 10 (CXCL10; or IP-10) protein had the highest fold change (> 10.0) in both DM and JDM patients compared to matched healthy controls (Supplementary Table 1). Seven other proteins had a fold change > 3.0 in DM patients compared to their matched healthy controls: S100 calcium binding protein A12 (S100A12; or ENRAGE), CXCL9 (MIG), nicotinamide phosphoribosyltransferase (NAMPT; or visfatin), C-C motif chemokine ligand 8 (CCL8; or MCP2), heat shock protein family A member 1 A (HSPA1A; or Hsp70), lactotransferrin (LTF) and S100A6 (alarmin). Two other proteins had a fold change > 3.0 in JDM patients compared to their matched healthy controls: matrix metallopeptidase 3 (MMP3), and growth differentiation factor 15 (GDF15).

### Co-regulated protein clusters

**Fig. 2:**
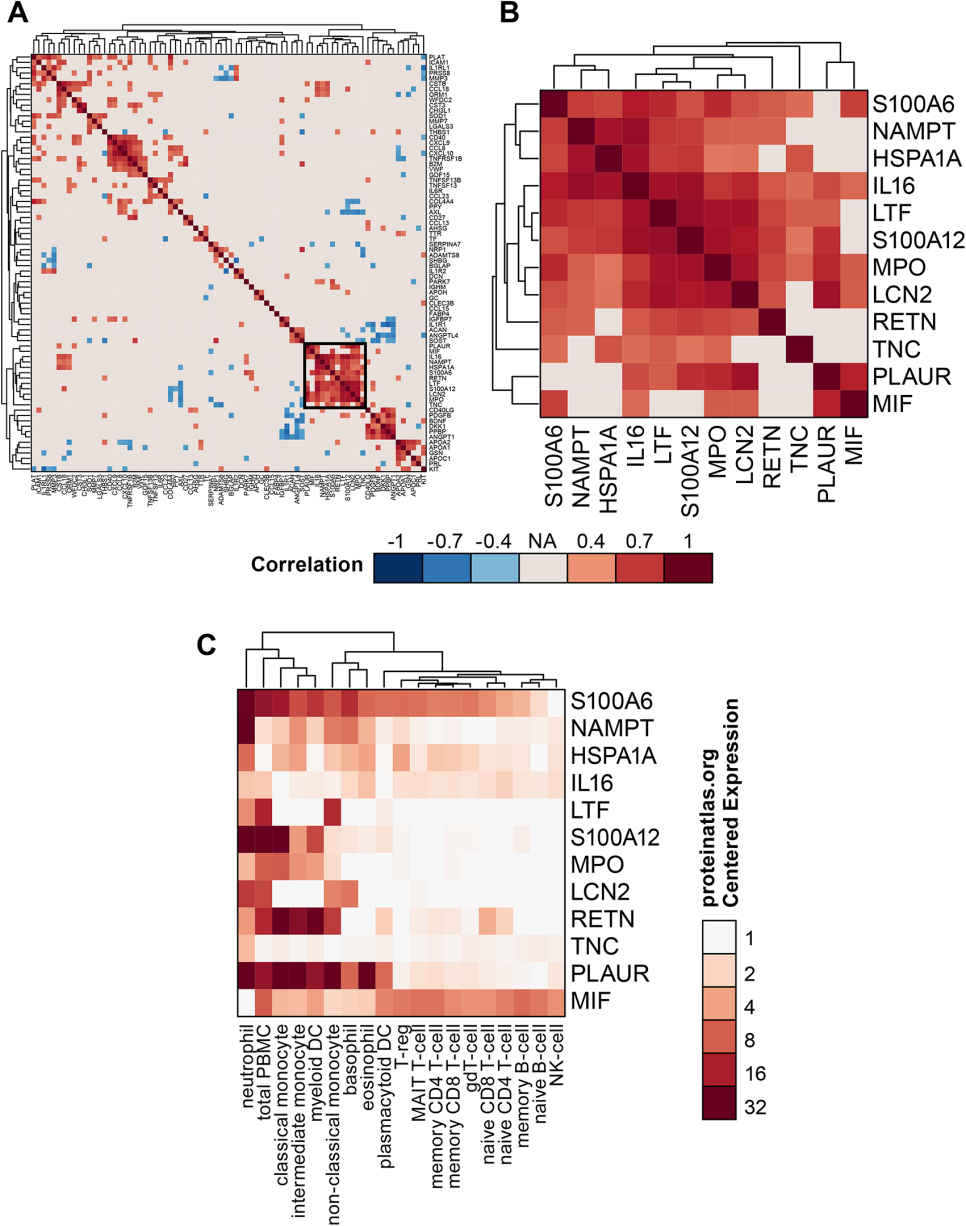
Protein-protein correlations in adult dermatomyositis (DM) patients identified a cluster associated with neutrophil, monocyte, and myeloid dendritic cell expression. (**A**) DM protein clustering of the 78 differentially expressed proteins. Correlations that met the thresholds of *p* < 0.05 and |Spearman’s rank correlation coefficient| > 0.4 were clustered. (**B**) The largest protein cluster, boxed in panel A, was further examined. (**C**) Relative expression levels of the clustered proteins in immune cell lineages. Abbreviations: DM, adult dermatomyositis; NA, not applicable

Multiple protein clusters with strong protein-protein correlations were visible in DM patients (Fig. 2A). The largest cluster consisted of 12 proteins (Fig. 2B): S100A6, NAMPT, HSPA1A, interleukin 16 (IL-16), LTF, S100A12, myeloperoxidase (MPO), lipocalin 2 (LCN2; or NGAL), resistin (RETN), tenascin C (TNC), urokinase receptor plasminogen activator (PLAUR; or uPAR), and macrophage migration inhibitory factor (MIF). These proteins were all upregulated in DM patients compared to their matched healthy controls. This cluster of proteins was associated with cell type-specific protein expression in neutrophils, monocytes, and myeloid DCs (mDCs) (Fig. 2C).

Multiple protein correlation clusters were visible in JDM patients (Supplementary Fig. 2A), the largest of which contained 14 proteins (Supplementary Fig. 2B). This cluster included some proteins in common with DM: HSPA1A, IL-16, LCN2, LTF, MIF, MPO, NAMPT, PLAUR, RETN, S100A6, and S100A12. Additional proteins in the cluster specific to JDM included: cystatin C (CST3), parkinsonism associated deglycase (PARK7), and superoxide dismutase 1 (SOD1). Of these proteins, only RETN was differentially expressed between JDM patients and their matched healthy controls (Supplementary Table 1). These proteins were also associated with cell type-specific protein expression in neutrophils, monocytes, mDCs, and plasmacytoid DCs (pDCs) (Supplementary Fig. 2C).

A cluster of IFN-associated proteins was apparent in both DM and JDM patients (Supplementary Fig. 3A, 3 C). Proteins present in both the DM and JDM clusters included beta-2 microglobulin (B2M), CCL8, CXCL9, CXCL10, tumor necrosis factor (TNF) receptor superfamily member 1B (TNFRSF1B), and GDF15. Proteins specific to the DM cluster included cluster of differentiation 40 (CD40) and von Willebrand factor (vWF) (Supplementary Fig. 3B). Proteins specific to the JDM cluster included CCL13, CCL15, intercellular adhesion molecule 1 (ICAM1), TNF superfamily member 13 (TNFSF13; or APRIL), and TNFSF13B (BAFF) (Supplementary Fig. 3D). All proteins in the DM IFN-associated cluster were upregulated in DM patients compared to their matched healthy controls, while in the JDM cluster, B2M, CXCL9, CXCL10, GDF15, and TNFSF13 were upregulated compared to matched healthy controls (Supplementary Table 1). All proteins in the DM cluster and the majority of proteins in the JDM cluster were associated with type I and II IFNs, except ICAM1 and CCL15, which were only associated with type II IFNs [25].

### Biological functions and upstream regulators

Pathway analysis was conducted independently for the differentially expressed proteins, 48 proteins in DM patients and 32 in JDM. The top associated pathways found in both DM and JDM included granulocyte and agranulocyte adhesion and diapedesis and pathogen induced cytokine storm signaling (Supplementary Table 2). Although these pathways were shared between DM and JDM, a number of the specific protein targets underlying the pathways were distinct, as indicated in the table. Pathways identified only in DM included Liver X Receptor/Retinoic X Receptor (LXR/RXR) Activation and 24-dehydrocholesterol reductase (DHCR24), and those identified exclusively in JDM included two osteoblast pathways (Supplementary Table 2).

These pathways are also represented in the top biologic functions, which are comprised of multiple pathways. The top biological functions identified in both DM and JDM were cellular movement, cell-to-cell signaling and interaction, and cell death and survival (Supplementary Table 3). Although these biological functions were shared between DM and JDM, many of the underlying target proteins differed between the two groups, as indicated in the table. Biological functions identified exclusively in DM included cell cycle and gene expression, while those identified only in JDM included cellular development and cellular function and maintenance (Supplementary Table 3).

Eighteen upstream regulators were identified in DM and four in JDM patients (Supplementary Table 4). Phosphoinositide 3-kinase (PI3K), p38 mitogen-activated protein kinase (MAPK), and toll-like receptor 7 (TLR7) were predicted to be activated in both DM and JDM. In DM, erythroblastic oncogene B (ERBB2; or HER2), IFN-gamma (IFNG), IL-18, IL-1A, IL-1B, IL-1R, MAPK8 (JNK1), nuclear factor kappa beta complex (NF-κB complex), non-POU domain containing octamer binding protein (NONO), RELA proto-oncogene NF-κB subunit (RELA), signal transducer and activator of transcription 1 (STAT1), TLR9, and TNF were also predicted to be activated. Estrogen receptor and interleukin enhancer binding factor 3 (ILF3) were predicted to be inhibited in DM. In JDM, STAT3 was predicted to be inhibited.

**Fig. 3:**
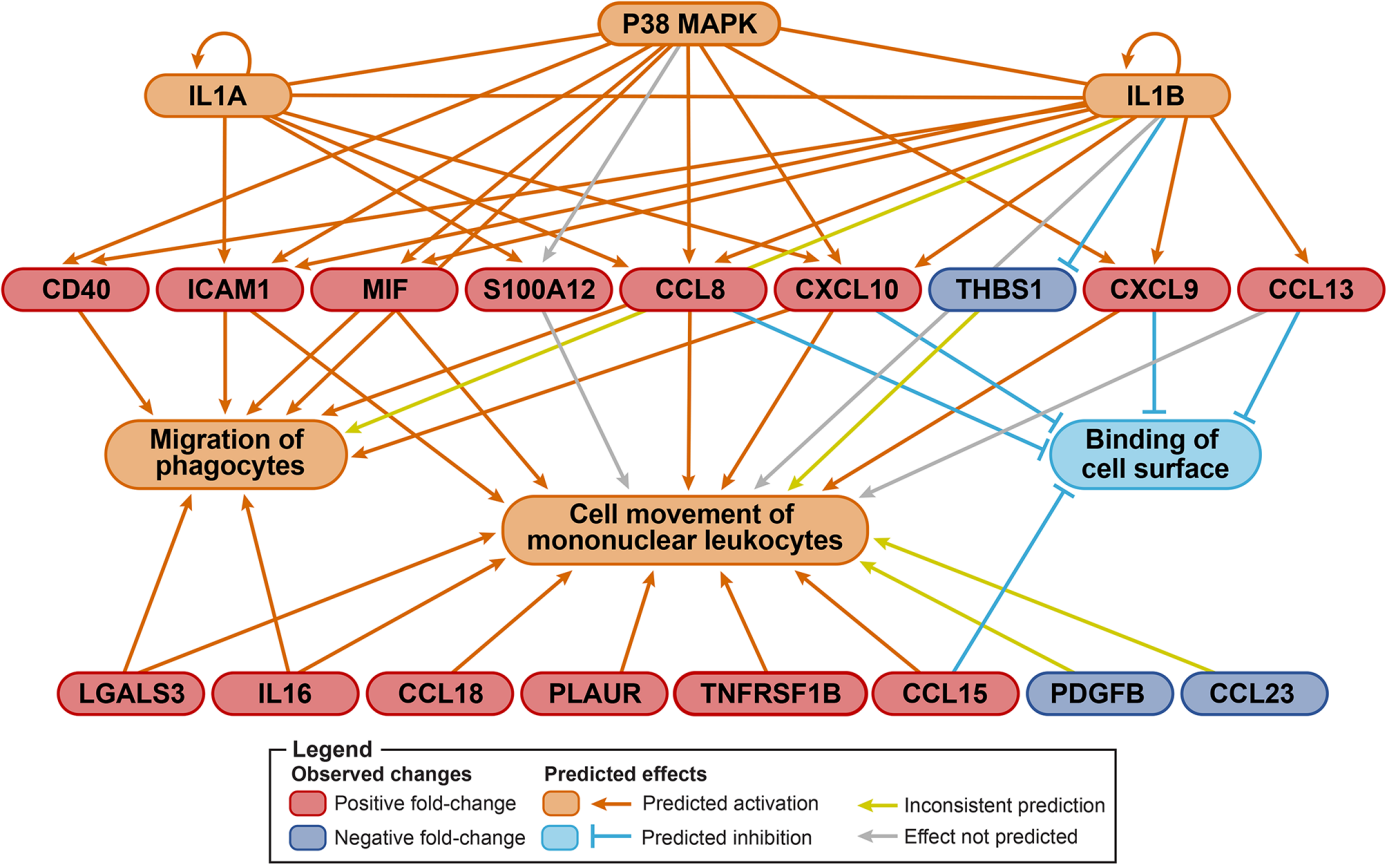
Differentially expressed proteins in adult dermatomyositis (DM) patients are involved in p38 MAPK signaling and immune cell movement. The top of the diagram depicts predicted upstream regulators, below which measured proteins are displayed in ovals. Below the measured proteins, predicted functional outputs of the measured proteins are displayed in three ovals. At the bottom of the diagram, measured proteins associated with the identified functions, but not the upstream regulators, are displayed. Orange boxes and lines indicate predicted activation; light blue boxes and lines indicate predicted inhibition; olive lines indicate the relationship is inconsistent with the state of the downstream molecule; gray lines indicate an association without predicted directionality. Red ovals indicate a positive fold change; dark blue ovals indicate a negative fold change. Abbreviations: DM, adult dermatomyositis

Fig. 3 depicts the upstream regulatory factors present in DM, based on the dysregulated proteins expressed in patient sera. p38 MAPK, IL-1A, and IL-1B were predicted to activate phagocyte migration and leukocyte movement, and to inhibit cell surface binding. The connection between the signaling pathways and phagocyte migration was found to be mediated by CD40, ICAM1, MIF, CCL8, and CXCL10. The connection between the signaling pathways and cell movement of mononuclear leukocytes was found to be mediated by ICAM1, MIF, S100A12, CCL8, CXCL10, thrombospondin 1 (THBS1), CXCL9, and CCL13 (MCP4). Finally, the connection between the signaling pathways and inhibition of cell surface binding was found to be mediated by CCL8, CXCL10, CXCL9, and CCL13. Additional proteins, such as galectin 3 (LGALS3) and IL-16, were associated with the identified functions but not with the upstream regulator.

### Protein correlation with disease activity measures

**Fig. 4:**
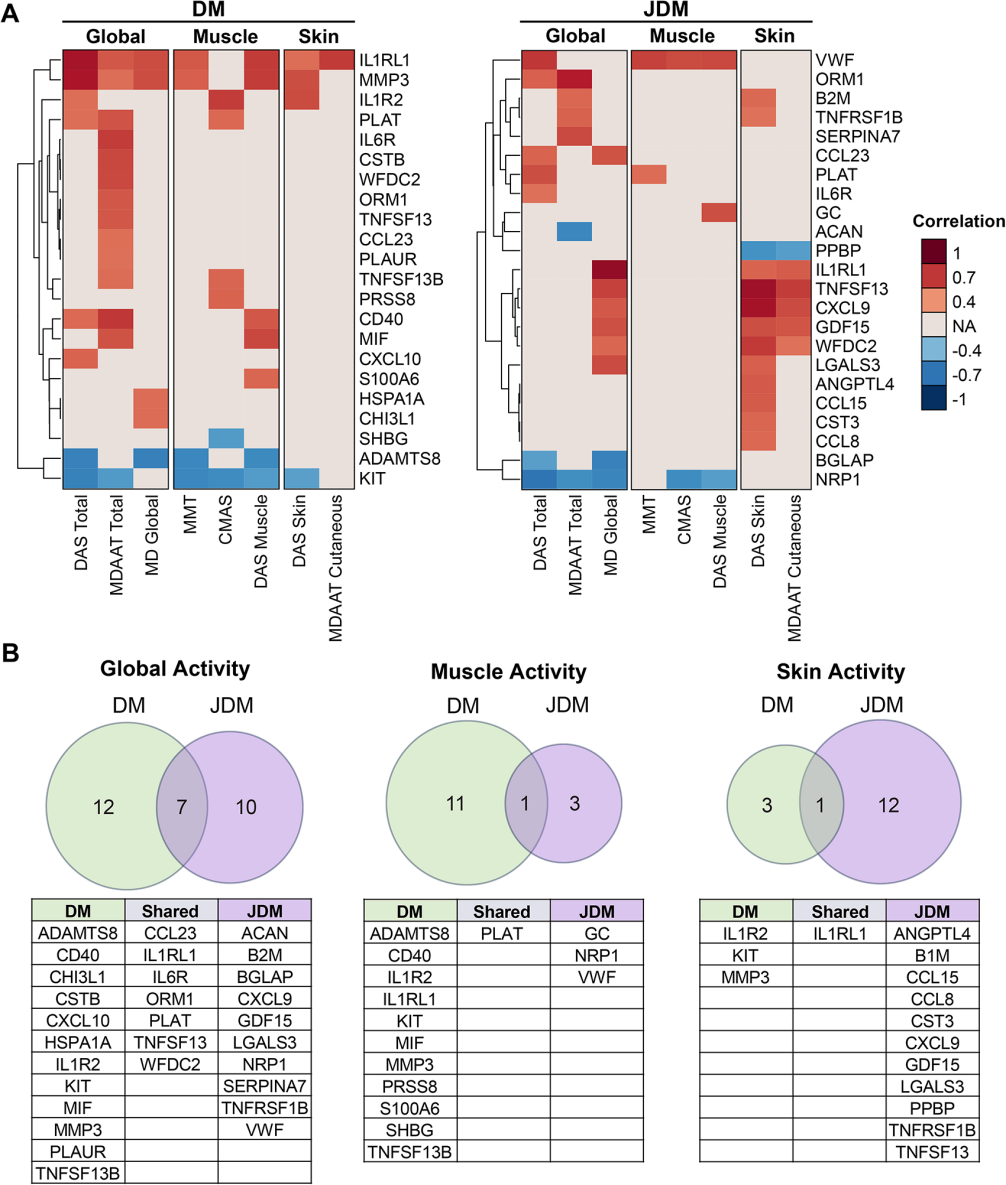
Correlations between differentially regulated proteins in adult dermatomyositis (DM) and juvenile dermatomyositis (JDM) patients and myositis disease activity measures. (**A**) Disease activity measures were categorized as measures of global, muscle, or skin disease activity. Correlations that met the thresholds of *p* < 0.05 and |Spearman’s rank correlation coefficient| > 0.4 are displayed. Red indicates a positive correlation; blue indicates a negative correlation. The left heatmap depicts DM correlations; the right heatmap depicts JDM correlations. (**B**) Comparisons of the proteins that correlated at least moderately with global, muscle, and skin disease activity in DM versus JDM patients. Shared and distinct correlated proteins from each category are tabulated below their respective Venn diagram. Abbreviations: DM, adult dermatomyositis; JDM, juvenile dermatomyositis; DAS, Disease Activity Score; MDAAT, Myositis Disease Activity Assessment Tool; MMT, Manual Muscle Testing; CMAS, Childhood Myositis Assessment Scale; NA, not applicable

Correlations between serum concentrations of the 78 differentially expressed proteins and disease activity measures were determined for DM and JDM patients (Fig. 4). Nineteen and 17 proteins correlated at least moderately with global disease activity in DM and JDM, respectively. Seven of these proteins were shared between DM and JDM, including IL-1 receptor like 1 (IL1RL1), orosomucoid 1 (ORM1), and tissue type plasminogen activator (PLAT; or tPA). Twelve and four proteins correlated at least moderately with muscle disease activity in DM and JDM, respectively. PLAT correlated with muscle disease activity in both DM and JDM. Finally, four and 13 proteins correlated at least moderately with skin disease activity in DM and JDM patients, respectively. IL1RL1 correlated with skin disease activity in both DM and JDM.

In DM, some proteins correlated with global, muscle, and skin disease activity, such as IL1RL1, MMP3, and tyrosine-protein kinase KIT (KIT). In JDM, no proteins correlated with global, muscle, and skin disease activity. Other proteins correlated with a specific disease activity category. In DM patients, IL-6R, cystatin B (CSTB), and WAP four-disulfide core domain 2 (WFDC2; or HE4) correlated with global disease activity, but not with muscle or skin disease activity. In JDM patients, pro-platelet basic protein (PPBP; or NAP2), IL1RL1, TNFSF13B, CXCL9, GDF15, and WFDC2 correlated with JDM skin disease activity, but not with muscle disease activity. Conversely, vWF correlated with JDM muscle disease activity, but not with skin disease activity. IL1RL1 correlated with multiple DM and JDM disease activity measures (Fig. 4).Significant correlations between RBM proteins and serum levels of muscle enzymes, including LDH, aldolase, and CK, are shown in Supplementary Fig. 4. Many of the proteins that correlated with muscle disease activity also correlated with serum muscle enzyme levels. For example, MMP3 and IL1RL1 correlated with muscle disease activity and also with muscle enzyme levels in DM. Similarly, PLAT and vWF correlated with muscle disease activity and muscle enzyme levels in JDM.

## Discussion

As part of a broader study examining the immunome of patients with DM and JDM [11], this study explored differentially regulated serum proteins in treated DM and JDM patients with persistently active disease. It validated previous reports of differentially regulated proteins, including CXCL10 and GDF15, and identified several new proteins associated with DM and JDM. The multiplex proteomic immunoassay used here allowed the study of multiple pathways in order to go beyond individual protein identification and instead characterize relationships among dysregulated proteins. Using the observed differentially regulated proteins, pathway analysis predicted upstream regulators, including PI3K, p38 MAPK, and TLR7. Protein-protein clustering analysis identified two notable subsets of tightly correlated proteins: one associated with neutrophils, monocytes, and DCs and another composed of IFN-associated proteins. Many of the differentially regulated proteins also correlated with global, muscle, and/or skin disease activity measures and may serve as candidate biomarkers of active disease. Most of these candidate disease activity biomarkers were distinct between DM and JDM; however, several were identified in both subgroups. These findings provide insight into the pathophysiology of DM and JDM and may inform the clinical assessment of patients with persistent disease.

Seventy-eight proteins were differentially regulated in the sera of DM and/or JDM patients compared to healthy controls; fourteen of these proteins were shared between the two subgroups. The shared proteins included several inflammatory factors previously reported to be elevated in DM and JDM patient sera: CXCL10 and CXCL9, cytokines that bind CXCR3 to promote immune cell activation and migration; GDF15, a cytokine involved in stress response pathways during cellular injury; MMP3, a matrix metalloproteinase associated with immune-mediated tissue injury; and vWF, a marker of endothelial damage [11, 28–36]. Other proteins exhibited dysregulation in only one subgroup. For example, proteins only changed in DM included a novel GC vitamin D binding protein (GC), which is associated with neutrophils, and the inflammatory markers NAMPT and S100A12 as previously reported [11, 37]. NAMPT has also been implicated in muscle homeostasis [38]. Proteins only changed in JDM included: fatty acid binding protein 4 (FABP4) and angiopoietin like 4 (ANGPTL4), both of which are involved in lipid metabolism; PLAT, involved in tissue remodeling and cell migration; collagen type IV alpha 4 chain (COL4A4), a component of basement membranes; and ORM1, associated with neutrophils. Each of these proteins represents a novel association with JDM, and together with the identification of GC in DM, they add insight into previously described mechanisms of DM and JDM pathogenesis, including roles for lipid metabolism, neutrophil regulation, and tissue remodeling [9–11, 39, 40].

Protein clustering and pathway analysis provided insight into the biological functions of the differentially regulated proteins. The largest protein cluster in DM and JDM was associated with mDCs, which have been shown to secrete type I IFNs in DM patients [41]. pDCs, which are potent secretors of type I IFNs, were also associated with the largest JDM protein cluster [42]. pDCs have been described as mediators of muscle and skin inflammation in untreated JDM patients, and their identification in the present study may reflect treatment-resistant disease activity [43, 44]. The presence of a smaller IFN-associated protein cluster in DM and JDM validates many studies, which have identified IFN signatures in the peripheral blood and affected tissues of DM and JDM patients [4, 6–8].

Additionally, granulocyte adhesion and diapedesis was a top associated pathway in both DM and JDM. Granulocyte signaling was further supported by protein-protein cluster analysis, which identified a group of proteins expressed by neutrophils and monocytes in both DM and JDM patients. These findings build upon previous studies that described upregulated neutrophil degranulation pathways and suggested that neutrophil dysregulation directly contributes to tissue damage in DM and JDM [9–11, 39, 40]. DM and JDM protein changes were also both enriched in pathogen-induced cytokine storm signaling. This finding expands upon previous reports showing elevated cytokines in DM patients, and notably that the DM cytokine signature mirrored that of severe COVID-19 cases [11, 45, 46].

Osteoblast pathways were uniquely observed in JDM, interesting in part because calcinosis is more common in JDM than DM [47]. While osteoblast activity has been implicated in other diseases involving calcification, only a few studies have associated osteoblasts specifically with calcinosis [48, 49]. For example, one group reported osteoblast markers near calcium deposits in JDM, although osteoblasts themselves were not observed near or in calcinosis lesions [50]. Additional studies are needed to assess a mechanistic connection related to osteoblast pathways in JDM, and whether this correlates with calcinosis in JDM patients.

Upstream regulatory analysis, based on the differentially expressed proteins, predicted activation of PI3K, p38 MAPK, and TLR7 signaling in both DM and JDM, which validated previous reports [11, 51]. Activated upstream regulators predicted only in DM included proinflammatory cytokines (IL-1A, IL-1B, IL-18, TNF), transcription factors (NF-κB), signaling mediators (MAPK8, STAT1), and innate immune receptors (IL-1R, TLR9), also supporting previous studies [46, 52–58]. Newly identified upstream regulators predicted only in DM included the activation of tyrosine kinase ERBB2 (HER2), and the inhibition of RNA binding protein ILF3. Predicted activation of ERBB2 only in DM is of interest given that DM, and not JDM, is associated with an increased risk of malignancy [2, 59], and ERBB2 over-expression is also associated with malignancies in numerous cancer types [60], although this needs further investigation in DM.

Predicted upstream regulators unique to JDM included inhibition of STAT3. Previous studies showed activated STAT3 signaling in DM, and increased STAT3 expression in DM compared to JDM [11, 61, 62]. STAT3 is a transcription factor with broad roles in inflammation and immunity [63]. Its role as a master regulator of Th17 development has been implicated in the pathogenesis of autoimmune diseases [64]. Given that elevated levels of Th17 cells have also been observed in DM compared to JDM [65], STAT3 signaling may reflect differences in the signaling pathways underlying DM and JDM and should be further studied.

Proteins were correlated with clinical disease activity measures to identify candidate biomarkers of disease activity. Thirty-nine of the 78 differentially regulated proteins strongly correlated with disease activity in DM, JDM, or both groups. Among these proteins, IL1RL1, an IL-33 receptor that stimulates MAPK signaling, emerged as a candidate global disease activity biomarker in both DM and JDM. In the present study, MAPK signaling was predicted to be activated in DM and JDM which has also been observed in previous studies [11, 66]. However, IL1RL1 has not been widely described as a DM or JDM biomarker; one report showed a positive correlation between serum IL1RL1 and DM global disease activity [67] and another group reported a strong positive correlation between serum IL1RL1 and disease activity in idiopathic inflammatory myopathies [68]. Further investigation of IL1RL1 as a candidate biomarker for DM and JDM global disease activity is needed.

Other candidate biomarkers correlated with specific target organs and/or subgroups. For example, MMP3 correlated well with DM, but not JDM, disease activity categories, while vWF was a good marker of JDM muscle disease activity. Additional candidate JDM skin disease activity biomarkers included the proinflammatory cytokines CXCL9, GDF15, and TNFSF13, the protease inhibitor WFDC2, and CST3. Although CST3 (cystatin C) is often used as a biomarker of renal function, it is also expressed by conventional type 1 DCs (cDC1s), which may relate to its correlation with JDM skin disease activity [69]. A greater number of proteins correlated with skin disease activity in JDM than DM, 14 versus four, and JDM is more often associated with refractory cutaneous disease [59]. Candidate biomarkers CXCL9, GDF15, and vWF have previously been shown to correlate with global JDM activity [14, 70, 71], and WFDC2 and TNFSF13 have been associated with interstitial lung disease in DM [72, 73].

Several novel candidate disease activity biomarkers were identified. The validation of previously described disease activity biomarkers, such as GDF15 and vWF, strengthens the finding of these novel candidates [14, 70, 71]. The acute-phase reactant ORM1 correlated with DM and JDM global disease activity. MMP3 and KIT, a receptor tyrosine kinase involved in PI3K and MAPK signaling, correlated with DM global, muscle, and skin disease activity. A disintegrin and metalloprotease with thrombospondin motifs 8 (ADAMTS8), and MIF, a proinflammatory cytokine that regulates macrophage activity, correlated with DM global and muscle disease activity. The regulatory T-cell (Treg) marker neuropilin 1 (NRP1) negatively correlated with JDM global and muscle disease activity. Further studies are needed to determine whether these novel candidate biomarkers are seen in other myositis populations and how they change in relation to changes in disease activity.

The multiplex proteomic immunoassay was a strength of the present study. By measuring over 200 proteins concurrently, the platform validated transcriptional studies and identified novel differential regulation at the protein level. Further, it extended beyond singular biomarker identification and permitted the identification of functions, pathways, and cell types associated with the collective changes. The RBM platform demonstrated specific advantages over other proteomic assays. For example, nearly 30% of the proteins measured in this study showed significant dysregulation in DM or JDM, a higher discovery rate than previous protein array results [74, 75]. Among these discoveries were several proteins not measured by previous protein arrays: ADAMTS8, apolipoprotein A2 (APOA2), APOH, AXL receptor tyrosine kinase (AXL), CD40, C-type lectin domain family 3 member B (CLEC3B), COL4A4, FABP4, GC, ORM1, serine protease 8 (PRSS8; or prostasin), TNFSF13, transthyretin (TTR), and WFDC2. Thus, this immunoassay provided an effective, focused platform for assessing DM and JDM protein dysregulation.

Limitations of the study include its small sample size, with inclusion of only 17 patients each for DM and JDM. This did not allow for examination of variations among myositis autoantibody (MAA) groups. Given that different MAAs are associated with different clinical features, it is possible that the present findings are not generalizable to all MAA groups and that some MAA-specific results were not detected [76]. Future studies with larger cohorts should conduct MAA subgroup analyses. While this study examined muscle and skin disease activity, lung disease activity has also been associated with adult DM [77]. The limited number of patients with lung involvement in this study prevented analysis of the correlation between serum proteins and lung disease activity. Future research should address this topic. Further, samples were available from only one timepoint, preventing examination of changes in disease activity. Nevertheless, statistical associations were strong, and the inclusion of both DM and JDM patients allowed for direct comparison of the two groups. Additionally, study participants were receiving medications, which may have prevented the identification of additional important pathways and biomarkers. However, this study specifically targeted the pathways and functions that persist despite treatment and may represent important targets for therapeutic intervention in chronically active patients. Finally, only serum protein concentrations were assessed, which may not represent dysregulated pathways at the site of tissue-specific pathology [78]. However, previous studies have described a strong relationship between expression in circulating blood and muscle biopsies [79, 80].

## Conclusions

In conclusion, the current study characterized similarities and differences in the pathophysiology of DM and JDM in the context of persistently active disease. The findings expand previous reports of the importance of IFN pathways, neutrophils, and mononuclear leukocytes in DM/JDM disease activity and add evidence to the role of key upstream regulators, including PI3K, p38 MAPK, and TLR7. Further, a number of candidate protein biomarkers of disease activity were identified. The many differentially regulated proteins, pathways, and biomarkers that were uniquely identified in DM or JDM suggest differences in the pathophysiology of DM and JDM. These proteins, pathways, and biomarkers should be further studied to better understand their roles in DM and JDM pathogenesis, and to further inform the clinical assessment and potential treatment targets in refractory patients.

## Electronic supplementary material

Below is the link to the electronic supplementary material.

Supplementary Material 1: Additional File 1: Supplementary Table 1. Fold changes, clinical correlations, and descriptions of the 78 differentially regulated proteins in adult dermatomyositis (DM) and juvenile dermatomyositis (JDM).

Supplementary Material 2: Additional File 2: Supplementary Figure 1. Differentially regulated proteins shared in both adult and juvenile dermatomyositis (DM, JDM). The Venn diagram depicts the overlap between the differentially regulated proteins in the DM (n = 48) and JDM (n = 32) analyses. Adult controls were used in the DM analysis and pediatric controls were used in the JDM analysis. Twelve additional proteins (not shown) were identified in the combined DM and JDM analysis, which included: ADAMTS8, APOC1, ANGPT1, B2M, CHI3L1, IGFBP7, IL1RL1, IL1R2, IL6R, PPY, RETN, and SERPINA7. Abbreviations: DM, adult dermatomyositis; JDM, juvenile dermatomyositis. Additional File 2: Supplementary Figure 2. Protein-protein correlations in juvenile dermatomyositis (JDM) patients associated with neutrophil, monocyte, and dendritic cell expression. (A) JDM protein clustering of the 78 differentially expressed proteins. Correlations that met the thresholds of p <0.05 and |Spearman’s rank correlation coefficient| > 0.4 were clustered. (B) The largest protein cluster, boxed in panel A, was further examined. (C) Relative expression levels of the clustered proteins in immune cells. Abbreviations: JDM, juvenile dermatomyositis; NA, not applicable. Additional File 2: Supplementary Figure 3. Protein-protein correlations in adult dermatomyositis (DM) and juvenile dermatomyositis (JDM) patients associated with interferons. (A) DM protein clustering of the 78 differentially expressed proteins. Correlations that met the thresholds of p <0.05 and |Spearman’s rank correlation coefficient| > 0.4 were clustered. (B) The second largest DM protein cluster, boxed in panel A, was further examined. (C) JDM protein clustering of the 78 differentially expressed proteins. Correlations that met the thresholds were clustered. (D) The second largest JDM protein cluster, boxed in panel C, was further examined. Abbreviations: DM, adult dermatomyositis; JDM, juvenile dermatomyositis; NA, not applicable. Additional File 2: Supplementary Figure 4. Significant correlations between serum muscle enzyme levels and proteins from the Rules-based Medicine platform in patients with adult dermatomyositis (DM) and juvenile dermatomyositis (JDM). Serum levels of lactate dehydrogenase (LDH), aldolase, and creatine kinase were correlated with proteins from the Rules-Based Medicine (RBM) proteomics platform. Correlations that met the thresholds of p <0.05 and |Spearman’s rank correlation coefficient| > 0.5 are displayed. Red indicates a positive correlation; blue indicates a negative correlation. (A) The left heatmap depicts correlations in patients with DM. (B) The right heatmap depicts correlations in patients with JDM. Proteins that were part of the 78 differentially expressed proteins are starred. RBM protein names are used in the heatmaps. Abbreviations: RBM, Rules-Based Medicine; DM, adult dermatomyositis; JDM, juvenile dermatomyositis; LDH, lactate dehydrogenase. Additional File 2: Supplementary Table 2. Top five canonical pathways associated with differentially expressed proteins in adult dermatomyositis (DM) and juvenile dermatomyositis (JDM). Pathways shared between DM and JDM are bolded. Shared proteins between DM and JDM in the same pathways are indicated in bold. Abbreviations: DM, adult dermatomyositis; JDM, juvenile dermatomyositis. Additional File 2: Supplementary Table 3. Top five molecular and cellular functions associated with differentially expressed proteins in adult dermatomyositis (DM) and juvenile dermatomyositis (JDM). Molecular and cellular functions shared between DM and JDM are bolded. Shared proteins between DM and JDM in the same molecular and cellular functions are indicated in bold. Abbreviations: DM, adult dermatomyositis; JDM, juvenile dermatomyositis. Additional File 2: Supplementary Table 4. Predicted upstream regulators of differentially expressed proteins in adult dermatomyositis (DM) and juvenile dermatomyositis (JDM). Upstream regulators shared between DM and JDM are bolded. Shared proteins between DM and JDM under the same upstream regulators are indicated in bold. Abbreviations: DM, adult dermatomyositis; JDM, juvenile dermatomyositis.

## Data Availability

The dataset supporting the conclusions of this article is available in the dbGaP repository, [accession phs003270.v2.p1].

## Abbreviations

ADAMTS8: A disintegrin and metalloprotease with thrombospondin motifs 8
ANGPTL4: Angiopoietin like 4
APO: Apolipoprotein
AXL: AXL Receptor tyrosine kinase
CCL: C-C motif chemokine ligand
CD40: Cluster of differentiation 40
CK: Creatine kinase
cDC1: Conventional type 1 dendritic cells
CLEC3B: C-type lectin domain family 3 member B
CMAS: Childhood Myositis Assessment Scale
COL4A4: Collagen type IV alpha 4 chain
CSTB: Cystatin B
CST3: Cystatin C
CXCL: C-X-C motif chemokine ligand
DAS: Disease Activity Score
DC: Dendritic cell
DHCR24: 24-Dehydrocholesterol reductase
DM: Dermatomyositis
ERBB2: Erythroblastic oncogene B
EULAR-ACR: European Alliance of Associations for Rheumatology – American College of Rheumatology
FABP4: Fatty acid binding protein 4
GC: GC vitamin D binding protein
GDF15: Growth differentiation factor 15
HSPA1A: Heat shock protein family A member 1 A
ICAM1: Intercellular adhesion molecule 1
IFN: Interferon
IFNG: Interferon-gamma
IL: Interleukin
IL1RL1: Interleukin like 1 receptor like 1
ILF3: Interleukin enhancer binding factor 3
IMACS: International Myositis Assessment and Clinical Studies
IVIg: Intravenous immunoglobulin
JDM: Juvenile dermatomyositis
KIT: Tyrosine-protein kinase KIT
LCN2: Lipocalin 2
LDH: Lactate dehydrogenase
LGALS3: Galectin 3
LTF: Lactoferrin
LXR/RXR: Liver X Receptor/Retinoic X Receptor
MAPK: Mitogen-activated protein kinase
MDAAT: Myositis Disease Activity Assessment Tool
mDC: Myeloid dendritic cell
MIF: Macrophage migration inhibitory factor
MMP3: Matrix metallopeptidase 3
MMT: Manual Muscle Testing
MPO: Myeloperoxidase
MSA: Myositis-specific autoantibody
NAMPT: Nicotinamide phosphoribosyltransferase
NET: Neutrophil extracellular trap
NF-κB: Nuclear factor kappa B
NIH: National Institutes of Health
NONO: Non-POU domain containing octamer binding protein
NRP1: Neuropilin 1
ORM1: Orosomucoid 1
PARK7: Parkinsonism associated deglycase
pDC: Plasmacytoid dendritic cell
PI3K: Phosphoinositide 3-kinase
PLAT: Plasminogen activator, tissue type
PLAUR: Urokinase receptor plasminogen activator
PPBP: Pro-platelet basic protein
PRSS8: Serine protease 8
RELA: RELA proto-oncogene NF-κB subunit
RETN: Resistin
RBM: Rules-Based Medicine
S100: S100 calcium binding protein
SOD1: Superoxide dismutase 1
STAT: Signal transducer and activator of transcription
THBS1: Thrombospondin 1
TLR: Toll-like receptor
TNC: Tenascin C
TNF: Tumor necrosis factor
TNFSF: Tumor necrosis factor superfamily member
TNFRSF: Tumor necrosis factor receptor superfamily member
Treg: Regulatory T-cell
TTR: Transthyretin
VAS: Visual analog scale
vWF: Von Willebrand factor
WFDC2: WAP four-disulfide core domain 2
