# Supplementary material for: Neutrophil and mononuclear leukocyte pathways and upstream regulators revealed by serum proteomics of adult and juvenile dermatomyositis
Source: Arthritis Res Ther. 2024 Nov 11;26:196. doi: 10.1186/s13075-024-03421-7 (PMC11552237; doi:10.1186/s13075-024-03421-7)
Supplement: Supplementary file 2 — Supplementary Material 2: Additional File 2: Supplementary Figure 1. Differentially regulated proteins shared in both adult and juvenile dermatomyositis (DM, JDM). The Venn diagram depicts the overlap between the differentially regulated proteins in the DM (n = 48) and JDM (n = 32) analyses. Adult controls were used in the DM analysis and pediatric controls were used in the JDM analysis. Twelve additional proteins (not shown) were identified in the combined DM and JDM analysis, which included: ADAMTS8, APOC1, ANGPT1, B2M, CHI3L1, IGFBP7, IL1RL1, IL1R2, IL6R, PPY, RETN, and SERPINA7. Abbreviations: DM, adult dermatomyositis; JDM, juvenile dermatomyositis. Additional File 2: Supplementary Figure 2. Protein-protein correlations in juvenile dermatomyositis (JDM) patients associated with neutrophil, monocyte, and dendritic cell expression. (A) JDM protein clustering of the 78 differentially expressed proteins. Correlations that met the thresholds of p <0.05 and |Spearman’s rank correlation coefficient| > 0.4 were clustered. (B) The largest protein cluster, boxed in panel A, was further examined. (C) Relative expression levels of the clustered proteins in immune cells. Abbreviations: JDM, juvenile dermatomyositis; NA, not applicable. Additional File 2: Supplementary Figure 3. Protein-protein correlations in adult dermatomyositis (DM) and juvenile dermatomyositis (JDM) patients associated with interferons. (A) DM protein clustering of the 78 differentially expressed proteins. Correlations that met the thresholds of p <0.05 and |Spearman’s rank correlation coefficient| > 0.4 were clustered. (B) The second largest DM protein cluster, boxed in panel A, was further examined. (C) JDM protein clustering of the 78 differentially expressed proteins. Correlations that met the thresholds were clustered. (D) The second largest JDM protein cluster, boxed in panel C, was further examined. Abbreviations: DM, adult dermatomyositis; JDM, juvenile dermatomyositis; NA, not applicable. Addit [file 13075_2024_3421_MOESM2_ESM.pdf]

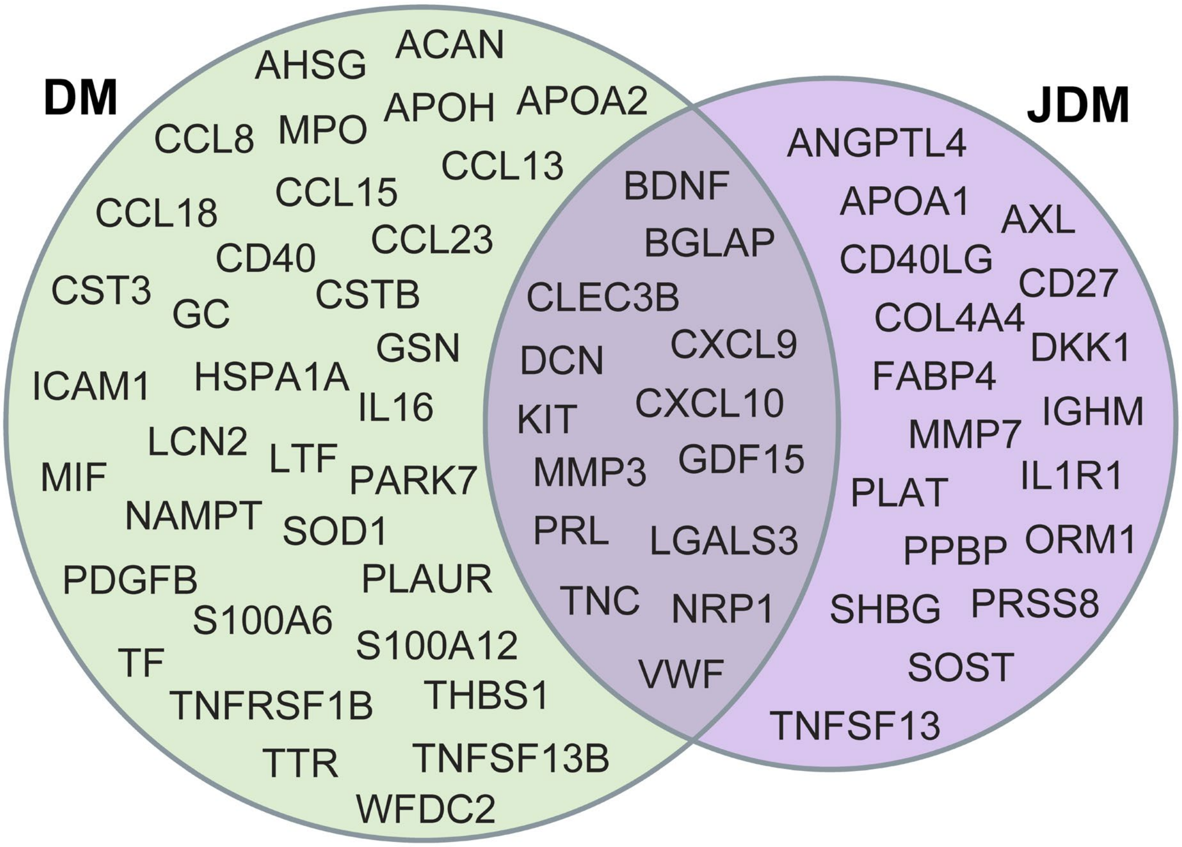

Supplementary Figure 1. Differentially regulated proteins shared in both adult and juvenile dermatomyositis (DM, JDM). The Venn diagram depicts the overlap between the differentially regulated proteins in the DM (n = 48) and JDM (n = 32) analyses. Adult controls were used in the DM analysis and pediatric controls were used in the JDM analysis. Twelve additional proteins (not shown) were identified in the combined DM and JDM analysis, which included: ADAMTS8, APOC1, ANGPT1, B2M, CHI3L1, IGFBP7, IL1RL1, IL1R2, IL6R, PPY, RETN, and SERPINA7. Abbreviations: DM, adult dermatomyositis; JDM, juvenile dermatomyositis.

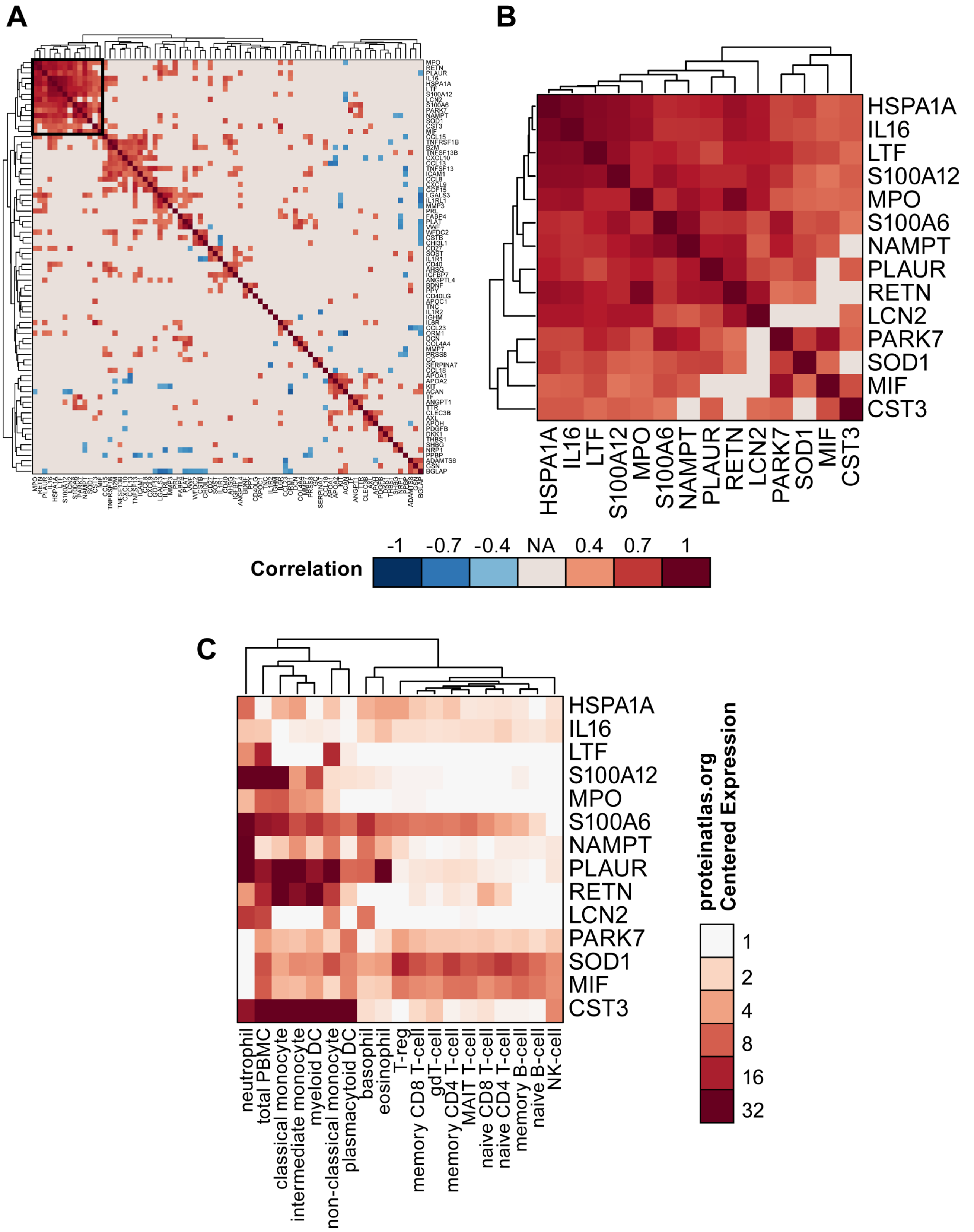

Supplementary Figure 2. Protein-protein correlations in juvenile dermatomyositis (JDM) patients associated with neutrophil, monocyte, and dendritic cell expression.

(**A**) JDM protein clustering of the 78 differentially expressed proteins. Correlations that met the thresholds of p <0.05 and |Spearman's rank correlation coefficient| > 0.4 were clustered. (**B**) The largest protein cluster, boxed in panel A, was further examined. (**C**) Relative expression levels of the clustered proteins in immune cells.

Abbreviations: JDM, juvenile dermatomyositis; NA, not applicable.

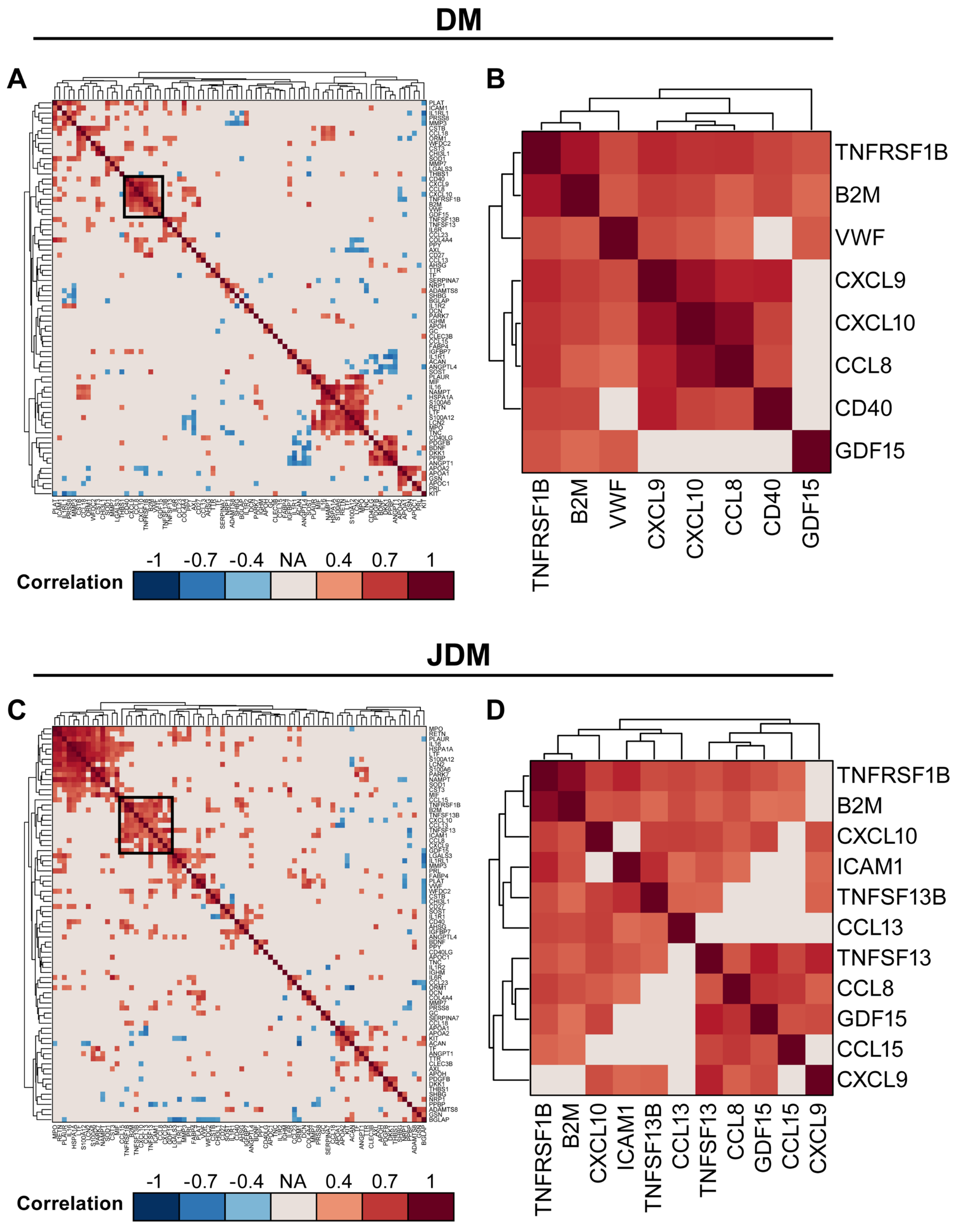

Supplementary Figure 3. Protein-protein correlations in adult dermatomyositis (DM) and juvenile dermatomyositis (JDM) patients associated with interferons.
(**A**) DM protein clustering of the 78 differentially expressed proteins. Correlations that met the thresholds of p <0.05 and |Spearman's rank correlation coefficient| > 0.4 were clustered. (**B**) The second largest DM protein cluster, boxed in panel A, was further examined. (**C**) JDM protein clustering of the 78 differentially expressed proteins. Correlations that met the thresholds were clustered. (**D**) The second largest JDM protein cluster, boxed in panel C, was further examined. Abbreviations: DM, adult dermatomyositis; JDM, juvenile dermatomyositis; NA, not applicable.

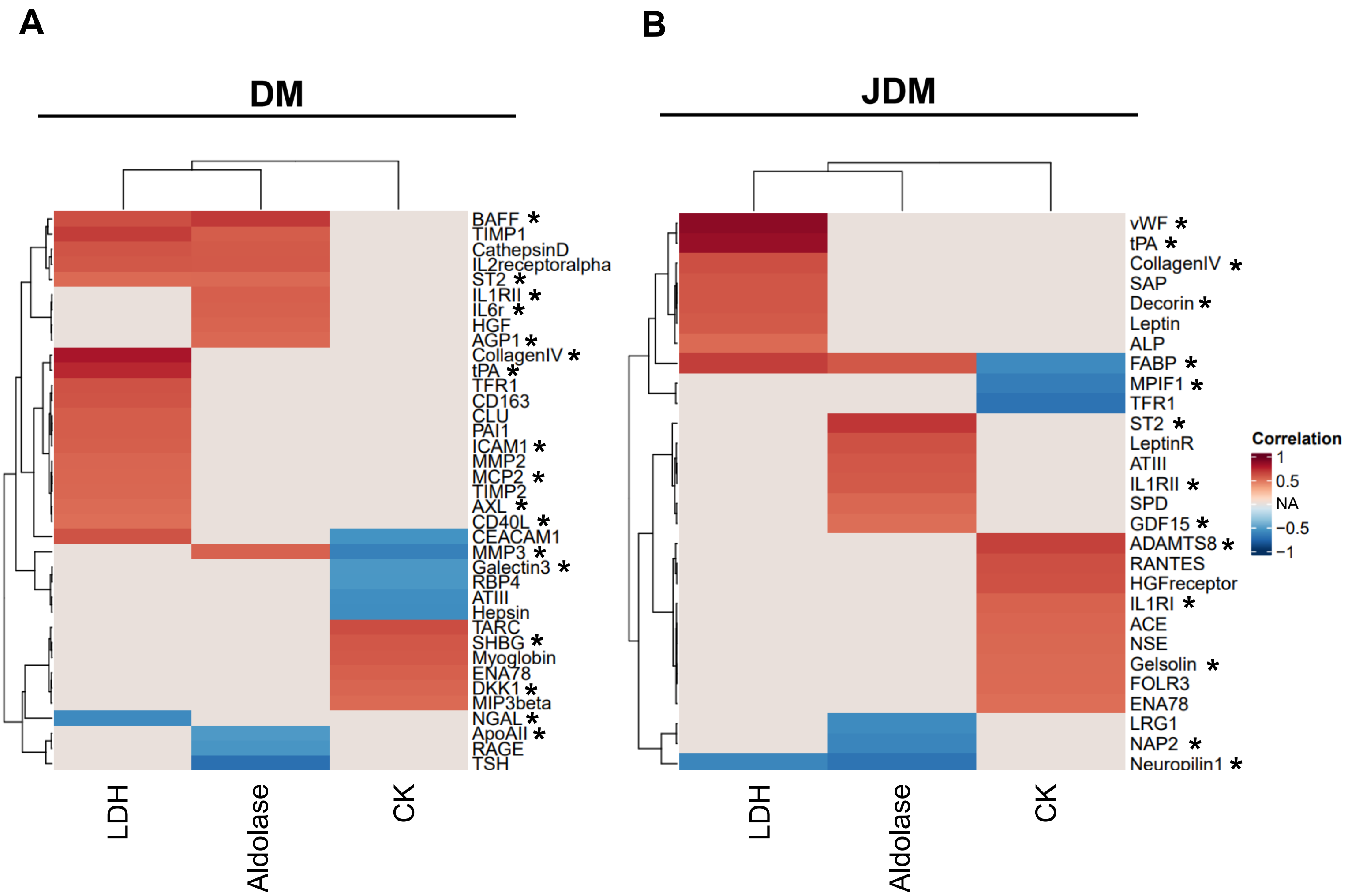

Supplementary Figure 4. Significant correlations between serum muscle enzyme levels and proteins from the Rules-based Medicine platform in patients with adult dermatomyositis (DM) and juvenile dermatomyositis (JDM).

Serum levels of lactate dehydrogenase (LDH), aldolase, and creatine kinase (CK) were correlated with proteins from the Rules-Based Medicine (RBM) proteomics platform. Correlations that met the thresholds of p <0.05 and |Spearman's rank correlation coefficient| > 0.5 are displayed. Red indicates a positive correlation; blue indicates a negative correlation. (A) The left heatmap depicts correlations in patients with DM. (B) The right heatmap depicts correlations in patients with JDM. Proteins that were part of the 78 differentially expressed proteins are starred. RBM protein names are used in the heatmaps.

Abbreviations: RBM, Rules-Based Medicine; DM, adult dermatomyositis; JDM, juvenile dermatomyositis; LDH, lactate dehydrogenase; CK, creatine kinase; NA, not applicable.

Supplementary Table 2: Top five canonical pathways associated with differentially expressed proteins in adult dermatomyositis (DM) and juvenile dermatomyositis (JDM).

Group	Associated Canonical Pathways*	-log(p-value)	Target Proteins†
DM	**Granulocyte Adhesion and Diapedesis**	11.400	CCL13, CCL15, CCL18, CCL23, CCL8, **CXCL10, CXCL9,** ICAM1, **MMP3,** TNFRSF1B
	**Agranulocyte Adhesion and Diapedesis**	9.510	CCL13, CCL15, CCL18, CCL23, CCL8, **CXCL10, CXCL9**, ICAM1, **MMP3**
	LXR/RXR Activation	8.320	AHSG, APOA2, APOH, GC, TF, TNFRSF1B, TTR
	DHCR24 Signaling Pathway	7.990	AHSG, APOA2, APOH, GC, PDGFB, TF, TTR
	**Pathogen Induced Cytokine Storm Signaling Pathway**	7.350	CCL13, CCL15, CCL18, CCL23, CCL8, **CXCL10, CXCL9**, TNFRSF1B, TNFSF13B
JDM	Role of Osteoblasts in Rheumatoid Arthritis Signaling Pathway	7.560	BGLAP, CD40LG, DKK1, MMP3, MMP7, SOST, TNFSF13
	**Granulocyte Adhesion and Diapedesis**	6.780	**CXCL10, CXCL9**, IL1R1, **MMP3**, MMP7, PPBP
	**Agranulocyte Adhesion and Diapedesis**	6.510	**CXCL10, CXCL9**, IL1R1, **MMP3**, MMP7, PPBP
	**Pathogen Induced Cytokine Storm Signaling Pathway**	6.320	CD40LG, COL4A4, **CXCL10, CXCL9**, IL1R1, PPBP, TNFSF13
	Role of Osteoblasts, Osteoclasts and Chondrocytes in Rheumatoid Arthritis	4.920	BGLAP, DKK1, IL1R1, MMP3, SOST

*Pathways shared between DM and JDM are indicated in bold
†Shared proteins between DM and JDM in the same pathways are indicated in bold

Supplementary Table 3: Top five molecular and cellular functions associated with differentially expressed proteins in adult dermatomyositis (DM) and juvenile dermatomyositis (JDM).

Group	Associated Molecular and Cellular Function*	Range of p-values	Target Proteins†
DM	**Cellular Movement**	4.82E-20 - 4.48E-03	**BDNF**, CCL13, CCL15, CCL18, CCL23, CCL8, CD40, CST3, **CXCL10**, **CXCL9**, **DCN**, GC, **GDF15**, GSN, HSPA1A/HSPA1B, ICAM1, IL16, **KIT**, LCN2, **LGALS3**, MIF, **MMP3**, NAMPT, **NRP1**, PARK7, PDGFB, PLAUR, **PRL**, S100A12, S100A6, THBS1, **TNC**, TNFRSF1B, **VWF**
	**Cell-To-Cell Signaling and Interaction**	1.2E-16 - 4.48E-03	AHSG, APOA2, APOH, **BDNF**, CCL13, CCL15, CCL18, CCL23, CCL8, CD40, CST3, **CXCL10**, **CXCL9**, **DCN**, GC, **GDF15**, GSN, HSPA1A/HSPA1B, ICAM1, IL16, **KIT**, **LGALS3**, LTF, MIF, NAMPT, **NRP1**, PDGFB, PLAUR, **PRL**, S100A12, S100A6, SOD1, THBS1, **TNC**, TNFRSF1B, TNFSF13B, **VWF**
	**Cell Death and Survival**	1.3E-10 - 4.48E-03	APOH, **BDNF**, CCL18, CD40, CST3, **CXCL10**, **CXCL9**, **GDF15**, GSN, HSPA1A/HSPA1B, ICAM1, **KIT**, LCN2, **LGALS3**, LTF, MIF, MPO, NAMPT, **NRP1**, PARK7, PDGFB, PLAUR, **PRL**, S100A6, SOD1, TF, THBS1, **TNC**, TNFRSF1B, TNFSF13B
	Cell Cycle	3.98E-09 - 4.48E-03	APOA2, APOH, CD40, DCN, GDF15, HSPA1A/HSPA1B, IL16, LGALS3, LTF, MIF, NRP1, PLAUR, PRL, SOD1, TF, TNC, TNFSF13B
	Gene Expression	3.98E-09 - 2.24E-03	APOH, CD40, GDF15, HSPA1A/HSPA1B, LTF, MIF, MPO, PLAUR, PRL, SOD1, TF, TNFSF13B
JDM	**Cell-To-Cell Signaling and Interaction**	1.12E-14 - 1.04E-02	ANGPTL4, APOA1, AXL, **BDNF**, CD27, CD40LG, **CXCL10**, **CXCL9**, **DCN**, **GDF15**, IGHM, IL1R1, **KIT**, **LGALS3**, MMP7, **NRP1**, ORM1, PLAT, PPBP, **PRL**, **TNC**, TNFSF13, **VWF**
	**Cellular Movement**	5.9E-12 - 8.94E-03	ANGPTL4, APOA1, AXL, **BDNF**, CD40LG, **CXCL10**, **CXCL9**, **DCN**, DKK1, GDF15, **KIT**, **LGALS3**, **MMP3**, MMP7, **NRP1**, ORM1, PLAT, PPBP, **PRL**, PRSS8, **TNC, VWF**
	**Cell Death and Survival**	2.55E-09 - 1.04E-02	ANGPTL4, APOA1, AXL, **BDNF**, CD27, CD40LG, **CXCL10**, **CXCL9**, DKK1, **GDF15**, IGHM, IL1R1, **KIT**, **LGALS3**, MMP7, **NRP1**, PLAT, PPBP, **PRL**, **TNC**, TNFSF13
	Cellular Development	5.67E-08 - 1.04E-02	ANGPTL4, APOA1, AXL, BDNF, CD27, CD40LG, CXCL10, CXCL9, DCN, DKK1, FABP4, GDF15, IGHM, IL1R1, KIT, LGALS3, MMP7, NRP1, PLAT, PRL, SHBG, SOST, TNC, TNFSF13
	Cellular Function and Maintenance	5.67E-08 - 7.46E-03	APOA1, AXL, BDNF, CD27, CD40LG, CXCL10, CXCL9, DCN, DKK1, FABP4, IGHM, KIT, LGALS3, NRP1, PLAT, PPBP, PRL, VWF

*Molecular and cellular functions shared between DM and JDM are indicated in bold

†Shared proteins between DM and JDM in the same molecular and cellular functions are indicated in bold

Supplementary Table 4: Predicted upstream regulators of differentially expressed proteins in adult dermatomyositis (DM) and juvenile dermatomyositis (JDM).

Group	Predicted Upstream Regulator*	Predicted Activation State	Activation z-score	-log(p-value of overlap)	Target Proteins†
DM	IL1B	Activated	2.781	10.578	ACAN, CCL13, CCL8, CD40, CXCL10, CXCL9, ICAM1, MIF, MMP3, NRP1, THBS1
	NFkB (complex)	Activated	2.762	9.845	CCL8, CD40, CXCL10, CXCL9, GDF15, ICAM1, KIT, LCN2, MMP3, NAMPT
	TNF	Activated	2.216	9.458	BDNF, CCL18, CD40, CXCL10, CXCL9, ICAM1, LCN2, LGALS3, MMP3, PLAUR, TNC, TNFRSF1B, TNFSF13B
	**P38 MAPK**	Activated	2.540	8.780	CCL8, CD40, **CXCL10, CXCL9,** ICAM1, MIF, **MMP3**, S100A12
	**PI3K (family)**	Activated	1.671	6.921	**CXCL10, CXCL9, MMP3,** PDGFB, THBS1, **TNC**
	IFNG	Activated	1.805	6.863	CCL18, CD40, CXCL10, CXCL9, HSPA1A/HSPA1B, ICAM1, THBS1, TNFRSF1B, TNFSF13B
	IL-1R	Activated	2.000	5.788	ICAM1, MMP3, PLAUR, TNFRSF1B
	Jnk	Activated	1.982	5.733	CD40, GDF15, ICAM1, MMP3, S100A12
	IL1A	Activated	2.190	5.710	CCL8, CXCL10, ICAM1, LCN2, S100A12
	ERBB2	Activated	1.982	5.503	CCL13, CCL18, CCL23, CXCL10, ICAM1, LCN2, PLAUR, THBS1
	IL18	Activated	1.955	4.900	CXCL10, CXCL9, ICAM1, MMP3
	TLR9	Activated	1.969	4.873	CD40, CXCL10, CXCL9, NAMPT
	**TLR7**	Activated	1.940	4.364	CD40, **CXCL10, CXCL9,** ICAM1
	RELA	Activated	1.969	3.785	CXCL10, CXCL9, ICAM1, KIT, NAMPT
	STAT1	Activated	1.981	3.693	CD40, CXCL10, CXCL9, ICAM1
	NONO	Activated	1.982	3.604	CCL8, CXCL10, CXCL9, TNFSF13B
	ILF3	Inhibited	-2.425	7.244	CCL23, CD40, CXCL10, LGALS3, PLAUR, TNC
	Estrogen Receptor	Inhibited	-2.000	2.939	HSPA1A/HSPA1B, ICAM1, PLAUR, TNC
JDM	**TLR7**	Activated	2.000	5.075	**CXCL10, CXCL9,** IL1R1, PLAT
	**PI3K (family)**	Activated	1.964	4.764	**CXCL10, CXCL9, MMP3, TNC**
	**P38 MAPK**	Activated	1.969	4.114	**CXCL10, CXCL9,** FABP4, **MMP3**
	STAT3	Inhibited	-1.732	3.365	ANGPTL4, CXCL10, CXCL9, DKK1

*Upstream regulators shared between DM and JDM are indicated in bold
†Shared proteins between DM and JDM under the same upstream regulators are indicated in bold
